# Comparative Genomics of *Escherichia coli* Sequence Type 219 Clones From the Same Patient: Evolution of the IncI1 *bla*_CMY_-Carrying Plasmid *in Vivo*

**DOI:** 10.3389/fmicb.2018.01518

**Published:** 2018-07-09

**Authors:** Cheng-Yen Kao, Jenn-Wei Chen, Tsung-Lin Liu, Jing-Jou Yan, Jiunn-Jong Wu

**Affiliations:** ^1^Department of Biotechnology and Laboratory Science in Medicine, School of Biomedical Science and Engineering, National Yang Ming University, Taipei, Taiwan; ^2^Department of Microbiology and Immunology, College of Medicine, National Cheng Kung University, Tainan, Taiwan; ^3^Department of Biotechnology and Bioindustry Sciences, National Cheng Kung University, Tainan, Taiwan; ^4^Department of Pathology, Cheng Ching Hospital at Chung Kang, Taichung, Taiwan

**Keywords:** comparative genomics, *bla*_**CMY**_, conjugative plasmid, evolution, ertapenem

## Abstract

This study investigates the evolution of an *Escherichia coli* sequence type 219 clone in a patient with recurrent urinary tract infection, comparing isolate EC974 obtained prior to antibiotic treatment and isolate EC1515 recovered after exposure to several β-lactam antibiotics (ceftriaxone, cefixime, and imipenem). EC974 had a smooth colony morphology, while EC1515 had a rough colony morphology on sheep blood agar. RAPD-PCR analysis suggested that both isolates belonged to the same clone. Antimicrobial susceptibility tests showed that EC1515 was more resistant to piperacillin/tazobactam, cefepime, cefpirome, and ertapenem than EC974. Comparative genomic analysis was used to investigate the genetic changes of EC974 and EC1515 within the host, and showed three plasmids with replicons IncI1, P0111, and IncFII in both isolates. P0111-type plasmids pEC974-2 and pEC1515-2, contained the antibiotic resistance genes *aadA2, tetA*, and *drfA12*. IncFII-type plasmids pEC974-3 and pEC1515-3 contained the antibiotic resistance genes *bla*_TEM−1_, *aadA1, aadA22, sul3*, and *inuF*. Interestingly, *bla*_CMY−111_ and *bla*_CMY−4_ were found in very similar IncI1 plasmids that also contained *aadA22* and *aac(3)-IId*, from isolates EC974 (pEC974-1) and EC1515 (pEC1515-1), respectively. The results showed *in vivo* amino acid substitutions converting *bla*_CMY−111_ to *bla*_CMY−4_ (R221W and A238V substitutions). Conjugation experiments showed a high frequency of IncI1 and IncFII plasmid co-transference. Transconjugants and DH5α cells harboring *bla*_CMY_-_4_ or *bla*_CMY_-_111_ showed higher levels of resistance to ampicillin, amoxicillin, cefazolin, cefuroxime, cefotaxime, cefixime, and ceftazidime, but not piperacillin/tazobactam, cefpime, or ertapenem. All known genes (outer membrane proteins and extended-spectrum AmpC β-lactamases) involved in ETP resistance in *E. coli* were identical between EC974 and EC1515. This is the first study to identify the evolution of an IncI1 plasmid within the host, and to characterize *bla*_CMY−111_ in *E. coli*.

## Introduction

*Enterobacteriaceae* manifest resistance to third-generation cephalosporins by the production of extended-spectrum β-lactamases (ESBLs), chromosomal AmpC (cAmpCs), or plasmid-mediated AmpCs (pAmpCs) (Jacoby, 2009). However, AmpCs do not hydrolyze fourth-generation cephalosporins, such as cefepime (FEP) (Jacoby, 2009). Therefore, FEP is suggested for the treatment of infections caused by AmpC producers. Plasmid-mediated extended-spectrum AmpC β-lactamases (pESACs) with mutations in AmpC that enhance catalytic activity toward cefepime, derived from the most frequently detected pAmpC, *bla*_CMY−2_, have been identified (Nordmann and Mammeri, 2007; Doi et al., 2009; Jacoby, 2009). Doi et al. showed a reduced susceptibility to FEP among *Escherichia coli* clinical isolates producing novel variants of *bla*_CMY−2_, *bla*_CMY−33_, and *bla*_CMY−44_ (Doi et al., 2009). However, for these pESACs, structural information regarding their hydrolytic performance on different β-lactam antibiotics is yet to be characterized.

Among the β-lactams currently available, carbapenems are unique because they are relatively resistant to hydrolysis by most β-lactamases (Barry et al., 1985). Carbapenems have thus been considered as the last resort of drugs for treating infections caused by multi-drug resistant Gram-negative bacilli. Carbapenem resistance in *Enterobacteriaceae* can arise by several mechanisms, including mutations that alter the expression and/or function of porins, or overexpression of active efflux pumps, while the greatest concern has been placed on acquired transferable carbapenemases (Temkin et al., 2014). *bla*_OXA_-type (*bla*_OXA−48_ and *bla*_OXA−181_) or *bla*_NDM_ are the dominant carbapenemases found in *E. coli* (Nordmann et al., 2011; Temkin et al., 2014; Al-Agamy et al., 2017). Overproduction of class C β-lactamases, such as *bla*_CMY−2_, or ESBL, can also lead to carbapenem resistance in *E. coli*, when combined with impermeability caused by porin (OmpC or OmpF) loss (Ma et al., 2013). Moreover, Dupont et al. reported that a Gly-63-Val substitution in OmpR, which codes for a regulatory protein involved in the control of OmpC/OmpF porin expression, caused the ertapenem resistance in *E. coli* (Dupont et al., 2017).

During evolution, the ability of bacteria to adapt to various hosts and environments has been favored by the acquisition of DNA elements through horizontal gene transfer or spontaneous mutations (Frost et al., 2005; Ilyas et al., 2017). The accumulation of antibiotic resistance, loss of nonessential genes, metabolic alterations, and virulence factor attenuation all occur when pathogenic organisms adapt to the host (Viberg et al., 2017). Antibiotic exposures also facilitate the genomic evolution of antibiotic resistance-associated genes (Pires et al., 2015; Lin et al., 2016). Here, we report a clinical case that illustrates the dynamic nature of an IncI1 plasmid within the host, as *bla*_CMY−111_ was changed to *bla*_CMY−4_ in *E. coli*.

## Materials and methods

### Bacterial strains and culture conditions

The bacterial strains and plasmids used in this study are described in Table 1. On day 1, an *E. coli* isolate (EC974) isolated from a 76-year-old women with urinary tract infection (UTI) showed intermediate resistance to piperacillin/tazobactam (TZP) but was susceptible to cefpirome (CPO), cefepime (FEP), and ertapenem (ETP). Once admitted, she was empirically started on ceftriaxone (CRO). After a week, the therapy was switched to cefixime (CFM) and continued for 4 days. One hundred and six days later, the woman was hospitalized due to recurrent UTI and was treated with imipenem (IMP). Two days later, an *E. coli* strain (EC1515) showing resistance to TZP, CPO, FEP, and ETP was isolated. *E. coli* was grown on Luria-Bertani (LB) agar or in broth. Bacteria harboring antibiotic resistance determinants were grown in the presence of the appropriate antibiotics at the following concentrations: ampicillin (AMP, 100 μg/mL); rifampicin (RIF, 256 μg/mL). All strains were stored at −80°C in LB broth containing 20% glycerol until testing.

**Table 1:** Strains and plasmids used in this study.

**Strain or plasmid**	**Relevant genotype or description**	**Reference or source**
**STRAIN**		
EC974	TZP^I^, FEP^S^, CPO^S^, ETP^S^	This study
EC1515	TZP^R^, FEP^SDD^, CPO^R^, ETP^R^	This study
C600	RIF^R^, recipient for conjugation assay	Laboratory stock
EC974-TCG	C600 transconjugant contains plasmids, pEC974-1 and pEC974-3	This study
EC1515-TCG	C600 transconjugant contains plasmids, pEC1515-1 and pEC1515-3	This study
DH5α	F^−^Ψ *80dlacZΔM15 Δ(lacZYA-argF) U169 hsdR17 recA1 thi-1 relA1*	Laboratory stock
DH5α-*bla*_CMY−111_	DH5α contains pACYC177-*bla*_CMY−111_	This study
DH5α-*bla*_CMY−4_	DH5α contains pACYC177-*bla*_CMY−4_	This study
**PLASMID**		
pACYC177	A general cloning vector; AMP^R^	Invitrogen
pACYC177-*bla*_CMY−111_	pACYC177 contains the *bla*_CMY−111_; AMP^R^	This study
pACYC177-*bla*_CMY−4_	pACYC177 contains the *bla*_CMY−4_; AMP^R^	This study

*AMP, ampicillin; CPO, cefpirome; ETP, ertapenem; FEP, cefepime; RIF, rifampicin; TZP, piperacillin/tazobactam*.

*S, sensitive; SDD, susceptible-dose dependent; I, intermediate resistance; R, resistant*.

### Antibiotic susceptibility testing

Antibiotic susceptibility of the strains was determined by the disk diffusion method on Mueller-Hinton (MH) agar, based on the CLSI guidelines (Clinical and Laboratory Standards Institute, 2009). Susceptibility to AMP, amoxicillin (AMC), amikacin (AN), chloramphenicol (CAP), ceftazidime (CAZ), CFM, ciprofloxacin (CIP), cefmetazole (CMZ), cefpodoxime (CPD), CPO, CRO, cefuroxime (CXM), cefazolin (CZ), ETP, FEP, gentamycin (GN), IPM, lomefloxacin (LOM), levofloxacin (LVX), meropenem (MEM), tobramycin (NN), cotrimoxazole (SXT), tetracycline (TE), and TZP was done. *E. coli* ATCC 25922 and *Klebsiella pneumoniae* ATCC 700603 were used as quality control strains. The interpretation of susceptibility to these antibiotics was determined according to the recommendations of the Clinical and Laboratory Standards Institute (2015).

### Genome sequencing, assembly, annotation, and analysis

Genomic DNA for *E. coli* EC974 and EC1515 was prepared using the Qiagen DNeasy Blood and Tissue kit (California, USA), according to the manufacturer's instructions. The genomes of EC974 and EC1515 were sequenced using the PacBio RS II platform (Pacific Biosciences, USA). HGAP3 in the SMRT analysis (v2.3) was used to assemble the PacBio data with default parameters, except the genome size was set as 5.5 Mb (Kao et al., 2017). HGAP3 analysis gave seven contigs for both strains. Contigs with a low coverage (< 20% of the mean) and/or shorter than 20 kb were discarded. This resulted in five and four contigs for the strains EC974 and EC1515, respectively. The remaining shortest contig of the strain EC974 was found highly similar to segments of another larger contig and there was a sudden drop in read coverage, so that contig was discarded. We therefore reported one chromosome and three plasmids for both strains.

For each contig, we aligned the first and last 20,000 bp using BLAT (v35, option: minMatch = 1) (Kent, 2002). Nearly perfect matches of lengths 4.6–16.6 kb were found for all contigs, suggesting their circular nature. We noted that the first 520 bp of the second largest contig of strain EC974 did not align well to the tail of the contig. The perfect match of ~4 kb which followed suggested false assembly at the contig start. Therefore, the first 520 bp of the contig was trimmed. A similar observation was made for the second largest contig of strain EC1515, and the first 554 bp was trimmed.

To circularize the contigs, we trimmed redundant segments at both contig ends. Specifically, the mid-point of the nearly perfect match was determined and bases from either end to the mid-point were trimmed. In addition, we adjusted contig orientation and starting position to match those of a reference sequence. For each contig, a closest reference was found via alignment against the NCBI NR database using BLAST. For both strains, the closest references (i.e., with the largest query coverage) of the four contigs from large to small were the chromosome of *E. coli* strain O157:H16, *Salmonella* enterica strain BL10 plasmid (CP025339), *E. coli* strain EHS30-1 plasmid (KX772391), and *E. coli* plasmid pH1038-142 (KJ484634), respectively. Instead of using the chromosome of *E. coli* strain O157:H16 as reference, we used the strain O157:H7 as reference because it is one of the official reference genomes of *E. coli*. Note that for the smallest contig of each strain, we fixed the starting position as the 61370th base of the plasmid pH1038-142 because the first base of that plasmid was not detected in our contig.

Sequences were annotated at the RAST prokaryotic genome annotation server (http://rast.nmpdr.org/) (Aziz et al., 2008). Microbial genome BLAST was used for the genome sequence similarity searching of the *E. coli* complete genome database (https://blast.ncbi.nlm.nih.gov/Blast.cgi?PAGE_TYPE=BlastSearch&BLAST_SPEC=MicrobialGenomes). The multilocus sequence typing (MLST), ResFinder, and PlasmidFinder databases (http://www.genomicepidemiology.org/) were used to find the sequence type (ST), serotype, antibiotic resistance genes, and the plasmid types present in the isolates EC974 and EC1515 (Larsen et al., 2012; Zankari et al., 2012). Insertion sequences (ISs) were characterized using the IS Finder database (https://www-is.biotoul.fr/).

### Comparative genome analysis

We used the command dnadiff of MUMMER (v3.23, default parameters) (Pop et al., 2004) to compare genomes of the two *E. coli* strains. Qualities of assemblies at the loci of SNPs and small INDELs were examined and those with a quality ≥ 30 within the homopolymer stretch were further analyzed.

### Nucleotide sequence accession numbers

The complete genome sequences of the *E. coli* strains EC974 and EC1515 have been deposited in GenBank under the accession numbers CP021840 (EC974 chromosome), CP021841 (pEC974-1), CP021842 (pEC974-2), CP021843 (pEC974-3), CP021844 (EC1515 chromosome), CP021845 (pEC1515-1), CP021846 (pEC1515-2), and CP021847 (pEC1515-3).

### Conjugation experiments and plasmid analysis

The liquid mating-out assay was used to transfer *bla*_CMY_ genes from parental isolates (EC974 or EC1515) to RIF-resistant *E. coli* C600 as described previously (Kao et al., 2016). Transconjugants were selected on LB plates containing RIF (256 μg/mL) and CTX (2 μg/mL). Plasmid numbers in the parental strains and transconjugants were determined as described previously (Kado and Liu, 1981).

### Cloning of *bla*_CMY_ genes

*bla*_CMY−4_ and *bla*_CMY−111_ were cloned into pACYC177 and transformed into *E. coli* DH5α cells. In brief, the 2,184 bp *bla*_CMY_ fragment (contained a promoter region) obtained from EC974 and EC1515 plasmid DNA and PCR with primers *bla*_CMY−1_ (5′-TTGCCGGCCGGTGGGTCATCTCTTGCTA-3′) and *bla*_CMY−2_ (5′-TTGCCGGCCGGTCGAGTGCAATTACGTT-3′), was digested by *Nae*I and ligated to the plasmid pACYC177 (digested by *Psi*I) to generate plasmids pACYC177-*bla*_CMY−111_ and pACYC177-*bla*_CMY−4_, respectively, and then transformed into DH5α cells. Antibiotics CTX (2 μg/mL) combined with AMP (100 μg/mL) were used to select for the *bla*_CMY−4_ or *bla*_CMY−111_ producers.

## Results

### Antibiotic susceptibility and clonal relationship of EC974 and EC1515

After CRO, CFM, and IMP treatments, EC1515 was isolated from a patient with recurrent UTI infection. Compared to EC974 from the same patient, EC1515 was more resistant to TZP, FEP, CPO, and ETP, than EC974 (Table 2), and had a rough colony morphology (Figure S1). To investigate whether the patient was recurrently infected by the same *E. coli* clone, the clonal relationships between the two isolates were analyzed by RAPD-PCR with primer 1254 (Pacheco et al., 1998). The results showed highly polymorphic profiles, distinguishing individual *E. coli* isolates (Figure S2). EC974 and EC1515 showing identical RAPD-PCR profiles were considered to be the same clone (Figure S2).

**Table 2:** Antibiotic susceptibility of *E. coli* isolates EC974 and EC1515.

**Isolate**	**Date**	**Antibiotic susceptibility**																							
		**AMP**	**AMC**	**TZP**	**CZ**	**CXM**	**CMZ**	**CAZ**	**CRO**	**CPD**	**CFM**	**FEP**	**CPO**	**IPM**	**ETP**	**MEM**	**LVX**	**CIP**	**LOM**	**GN**	**NN**	**AN**	**TE**	**CAP**	**SXT**
EC974	960824	R	R	I	R	R	R	R	R	R	R	S	S	S	S	S	S	I	R	S	S	S	R	R	R
EC1515	961210	R	R	R	R	R	R	R	R	R	R	SDD	R	S	R	S	S	I	R	S	S	S	R	R	R

*Antibiotic susceptibility tests were performed in duplicate*.

*AMP, ampicillin; AMC, amoxicillin; TZP, piperacillin/tazobactam; CZ, cefazolin; CXM, cefuroxime; CMZ, cefmetazole; CAZ, ceftazidime; CRO, ceftriaxone; CPD, cefpodoxime; CFM, cefixime; FEP, cefepime; CPO, cefpirome; IPM, imipenem; ETP, ertapenem; MEM, meropenem; LVX, levofloxacin; CIP, ciprofloxacin; LOM, lomefloxacin; GN, gentamycin; NN, tobramycin; AN, amikacin; TE, tetracycline; CAP, chloramphenicol; SXT, cotrimoxazole*.

*S, sensitive; SDD, susceptible-dose dependent; I, intermediate resistance; R, resistant*.

### Complete genome sequences of EC974 and EC1515 and their characteristics

For the two *E. coli* strains EC974 and EC1515, PacBio sequencing generated 106,134 and 106,265 polymerase reads, and the total numbers of bases were 1,475,613,460 and 1,581,950,763, respectively. The N50 lengths of the 159,569 and 177,682 subreads were 12,365 and 12,090 bp, respectively. For each strain, the data was assembled using HGAP3 and the resulting contigs were circularized. Mean read coverage across the genome were 147X and 198X, respectively for strains EC974 and EC1515. We reported one chromosome and three plasmids for both strains and their lengths were shown in Table 3. The results showed that the EC974 and EC1515 genomes had a size of 5,168,339 and 5,167,364 bp, respectively (Table 3). The GC contents of the two strains were both 50.24% (Table 3). RAST annotation results showed that the EC974 genome contained 4,906 coding sequences and 112 RNA genes, and the EC1515 genome contained 4,911 coding sequences and 112 RNA genes (Table 3). Moreover, 61% of the protein coding sequences were functionally annotated by the RAST server into 610 and 609 subsystems in EC974 and EC1515 strains, respectively.

**Table 3:** Characteristics of chromosomes and plasmids in isolates EC974 and EC1515.

**Characteristics**						
**CHROMOSOMES**						
	**Size (bp)**	**GC contents (%)**		**ORF (*****n*****)**	**RNA (*****n*****)**	**Antimicrobial resistance genes**
EC974	5,168,339	50.24		4906	112	*bla*_TEM−1_, *cmlA1, sul3, tetM, mefB, aadA1*
EC1515	5,167,364	50.24		4911	112	*bla*_TEM−1_, *cmlA1, sul3, tetM, mefB, aadA1*
**PLASMIDS**						
	**Size (bp)**	**Replicon**	**ORF**	**Conjugative related genes**	**High similarity plasmid (gene bank accession)**	**Antimicrobial resistance genes**
EC974						
pEC974-1	105,321	IncI1	123	+	*Escherichia coli* strain TVGHEC01 plasmid (LC019731)	*aadA22, aac(3)-IId, bla*_CMY−111_
pEC974-2	92,404	P0111	105	–	*Escherichia coli* strain EHS30-1, plasmid pEHS30-1 (KX772391)	*aadA2, tetA, dfrA12*
pEC974-3	78,518	IncFII	94	+	*Escherichia coli* strain V423 plasmid pV423-b (LC056646)	*bla*_TEM−1_, *aadA1, aadA22, sul3, inuF*
EC1515						
pEC1515-1	105,492	IncI1	123	+	*Escherichia coli* strain TVGHEC01 plasmid (LC019731)	*aadA22, aac(3)-IId, bla*_CMY−4_
pEC1515-2	92,352	P0111	102	-	*Escherichia coli* strain EHS30-1, plasmid pEHS30-1 (KX772391)	*aadA2, tetA, dfrA12*
pEC1515-3	78,518	IncFII	92	+	*Escherichia coli* strain V423 plasmid pV423-b (LC056646)	*bla*_TEM−1_, *aadA1, aadA22, sul3, inuF*

Serotype was determined by the genotype of *fliC, wzx*, and *wzy*, and the results showed that both strains belonged to the O51-H34 serotype. Antimicrobial resistance genes against β-lactams (*bla*_TEM−1_), phenicol (*cmlA1*), sulphonamide (*sul3*), tetracycline (*tetM*), macrolide (*mefB*) and aminoglycoside (*aadA1*) were identified on both chromosome sequences (Table 3). The MLST scheme of both isolates based on the sequences of seven housekeeping genes (*gyrB53, recA42, mdh24, purA1, icd58, adk58, fumC53*) confirmed that both isolates belonged to the ST219 type. Microbial genome BLAST results showed that the genome sequence of EC974 was highly similar to EC1515 (coverage 100%, identity 99%), DSM 103246 (coverage 92%, identity 99%, ST773), Santai (coverage 90%, identity 99%, ST773), RM12579 (coverage 90%, identity 99%, ST553), 2013C-4465 (coverage 90%, identity 99%, ST553), and CB9615 (coverage 90%, identity 99%, ST553), among 1,593 *E. coli* genomes in the database.

We further compared the genomes of the two strains using MUMMER and showed the 1-to-1 alignments allowing rearrangements in Table 4. The results revealed that only two large-scale rearrangements on the chromosome: one 973 bp deletion (Chromosome: 941793-942765 of strain EC974) and one 1,881 bp inversion (Chromosome: 3045861-3047678 of strain EC974) on the chromosome. For the largest plasmid, we observed one 532 bp insertion (pEC1515-1: 17188-17719 of strain EC1515) and one 326 bp deletion (pEC974-1: 17647-17973 of strain EC974). No large-scale rearrangement was observed on the other two plasmids. The shortest plasmids of the two strains were perfectly identical. MUMMER also revealed 12 SNPs and 35 small INDELs. Of the 12 SNPs, 9 and 3 resided in the chromosome and plasmid 1, respectively (Table 5 and Table S1). Among the 12 SNPs, eight were missense mutations and the rest were either intergenic (three) or synonymous (one) (Table 5). A majority of small INDELs were likely false due to the error-prone nature of PacBio data. Consistently, we found that many small INDELs occurred at loci of homopolymers and the assembly qualities were low. Of the 35 small INDELs, only 8 were of a high quality (quality ≥ 30) (Table 5). Among the 8 high quality small INDELs, four resulted in a frame shift while the rest resided in intergenic regions (Table 5). Taken together, these results showed the evolutionary relationships between EC974 and EC1515.

**Table 4:** One-to-one alignments of the two genomes using MUMMER.

**Contig**	**EC974**		**EC1515**		**Identity (%)**
	**Start**	**End**	**Start**	**End**	
Chromosome	1	941792	1	941792	100.00
Chromosome	941792	3045882	940823	3044907	99.99
Chromosome	3045861	3047678	3046703	3044886	99.89
Chromosome	3047657	5168339	3046682	5167364	99.99
pEC974-1 & pEC1515-1	1	17197	1	17187	99.91
pEC974-1 & pEC1515-1	17205	17664	17720	18164	95.03
pEC974-1 & pEC1515-1	17974	105321	18145	105492	99.99
pEC974-2 & pEC1515-2	1	92404	1	92352	99.94
pEC974-3 & pEC1515-3	1	78518	1	78518	100.00

**Table 5:** High quality SNPs and small INDELs between the EC974 and EC1515 genomes.

**Contig**	**Strain 974**		**Strain 1515**		**Mutation type^a^**	**Gene product**
	**Locus**	**Base**	**Locus**	**Base**		
**SNPs**						
Chromosome	1378463	C	1377490	G	ns (A-P)	Putative transcriptional repressor
Chromosome	1588773	A	1587799	G	ns (M-V)	Outer membrane protein G precursor
Chromosome	1857685	C	1856711	T	s	Putative transport protein
Chromosome	2230098	T	2229124	G	nc	–
Chromosome	2414261	A	2413286	T	ns (Q-L)	Response regulator BaeR
Chromosome	2514423	G	2513448	C	nc	–
Chromosome	3073707	A	3072732	T	ns (D-E)	Primosomal protein I
Chromosome	3260965	G	3259990	A	ns (S-F)	Lactaldehyde reductase
Chromosome	4466967	G	4465993	T	ns (G-V)	ATP-dependent DNA helicase RecQ
pEC974-1 & pEC1515-1	1392	T	1392	A	nc	–
pEC974-1 & pEC1515-1	49297	T	49468	A	ns (W-R)	β-lactamase
pEC974-1 & pEC1515-1	49349	T	49520	C	ns (V-A)	β-lactamase
**INDELs**						
Chromosome	1524239	T	1523265	–	fs	Hypothetical protein
Chromosome	2352437	T	2351462	–	fs	Glycosyltransferase
pEC974-1 & pEC1515-1	16847	C	16842	–	fs	Conjugative transfer pilus-tip adhesin protein, PilV
pEC974-1 & pEC1515-1	17171	G	17160	–	nc	–
pEC974-1 & pEC1515-1	17173	A	17161	–	nc	–
pEC974-1 & pEC1515-1	17177	–	17166	T	nc	–
pEC974-1 & pEC1515-1	17178	–	17168	C	nc	–
pEC974-2 & pEC1515-2	36578-36629	CAGAAA TGCTCC ACGTAC TCGGCC CCTTCC GGCGTG AAGGCG GCATCG AGCG	36577	-	fs	Transposon Tn21 modulator

a *ns, non-synonymous; s, synonymous; nc, non-coding region; fs, frame-shift*.

According to our annotation, the large deleted segments on chromosome contained two mobile element genes. The left boundary of the inversion on chromosome does not break any gene, but the right one broke a gene coding for phage tail fiber protein. The inverted segments contained two genes for phage tail fiber assembly. On the largest plasmid, the insertion did not break any gene and no gene was annotated in the inserted segment. The deletion broke a gene coding for Incl1 plasmid conjugative transfer pilus-tip adhesin protein, PilV.

We next examined the genes associated with repair systems in EC974 and EC1515 to determine whether EC1515 was a hypermutator. The results showed that the base excision repair system (uracil-DNA glycosylase, endonuclease III), nucleotide excision repair system (UvrABC), mismatch repair (MutSLH), and repair related genes (*recA, recF, radA*, and *radC*) were identical in EC974 and EC1515. However, RecQ, an ATP-dependent DNA helicase involved in DNA repair, showed an amino acid substitution in EC1515 (Table 5).

The numbers of plasmid in isolates EC974 and EC1515 were verified by Kado-Liu's method (Figure S3). The results showed three plasmids with replicons IncI1, P0111, and IncFII were found in both isolates (Table 3). Moreover, a high similarity of plasmid sequences between EC974 and EC1515 was observed (Table 4). These results excluded the possibility of lateral acquisition of plasmids and indicated the evolution of plasmids within the host. Conjugation-related genes were identified in IncI1- and IncFII plasmids. P0111-type plasmids pEC974-2 and pEC1515-2, contained the antibiotic resistance genes *aadA2, tetA*, and *drfA12*, which confer resistance to aminoglycosides, tetracycline, and trimethoprim, respectively (Table 3). IncFII-type plasmids pEC974-3 and pEC1515-3 contained antibiotic resistance genes *bla*_TEM−1_, *aadA1, aadA22, sul3*, and *inuF*. Interestingly, *aadA22, aac(3)-IId*, and *bla*_CMY−111_ were found in IncI1-plasmid pEC974-1, and *aadA22, aac(3)-IId*, and *bla*_CMY−4_ were identified in pEC1515-1 (Table 3). *bla*_CMY−4_ was different from *bla*_CMY−111_ by W221R and V238A substitutions. Both *bla*_CMY_ genes were located downstream of an IS*Ecp1* (family, IS1380), an element commonly associated with *bla*_CMY_ genes (Seiffert et al., 2013).

### Resistance gene transfer and plasmid analysis

*bla*_CMY−111_ was first identified in *Serratia marcescens* strain A4Y201 (Boyd et al., 2015); however, the function of *bla*_CMY−111_ in conferring antibiotic resistance is still unclear. Transfer of antibiotic resistance phenotypes in pEC974-1 and pEC1515-1 was determined by conjugation tests, and the plasmid sizes present in parental isolates and transconjugants were verified by Kado and Liu's methods (Figure S3). We randomly selected 10 transconjugants derived from EC974 and EC1515, and the results revealed that all transconjugants contained two plasmids, in IncI1- and IncFII groups (Figure S3). Transconjugants harboring *bla*_CMY_-_4_ (EC1515-TCG) or *bla*_CMY_-_111_ (EC974-TCG) showed higher level resistance to AMP, AMC, CZ, CXM, CTX, CFM, and CAZ, but not TZP, FEP, or ETP (Table 6). We further cloned *bla*_CMY_-_4_ and *bla*_CMY_-_111_ genes into pACYC177 to verify their resistance phenotypes. Compared to the DH5α cells, DH5α-*bla*_CMY_-_4_ and DH5α-*bla*_CMY_-_111_ were highly resistant to AMP, AMC, CZ, CXM, CTX, CFM, and CAZ (Table 6). These results indicated that *bla*_CMY_-_4_ and *bla*_CMY_-_111_ showed similar antibiotic resistance characteristics.

**Table 6:** Antibiotic susceptibility of the *E. coli* strains.

**Strains**	**Antibiotic susceptibility (diameter, mm)**																
	**AMP**	**AMC**	**TZP**	**CZ**	**CXM**	**CAZ**	**CFM**	**CTX**	**FEP**	**IPM**	**ETP**	**MEM**	**LVX**	**CIP**	**GN**	**AN**	**SXT**
EC974	R (6)	R (7)	I (18)	R (6)	R (6)	R (6)	R (6)	R (6)	S (25)	S (25)	S (23)	S (30)	S (18)	I (17)	S (25)	S (24)	R (6)
EC1515	R (6)	R (6)	R (10)	R (6)	R (6)	R (6)	R (6)	R (6)	SDD (19)	S (26)	R (17)	S (29)	S (18)	I (17)	S (26)	S (26)	R (6)
C600	S (16)	S (23)	S (35)	S (31)	S (30)	S (36)	S (27)	S (42)	S (42)	S (36)	S (40)	S (42)	S (32)	S (35)	S (25)	S (26)	S (32)
EC974-TCG	R (6)	R (7)	S (22)	R (6)	R (6)	R (14)	R (6)	R (17)	S (35)	S (32)	S (31)	S (39)	S (33)	S (36)	S (27)	S (28)	S (28)
EC1515-TCG	R (6)	R (7)	S (26)	R (6)	R (6)	R (10)	R (6)	R (12)	S (35)	S (37)	S (35)	S (40)	S (36)	S (37)	S (28)	S (29)	S (29)
DH5α	S (20)	S (26)	S (36)	S (31)	S (30)	S (35)	S (30)	S (42)	S (40)	S (36)	S (45)	S (39)	S (32)	S (34)	S (27)	S (28)	S (37)
DH5α-pACYC177	R (6)	S (19)	S (30)	S (29)	S (33)	S (40)	S (34)	S (48)	S (41)	S (43)	S (40)	S (44)	S (37)	S (40)	S (30)	S (33)	S (45)
DH5α-*bla*_CMY−111_	R (6)	R (6)	S (26)	R (6)	R (6)	R (8)	R (6)	R (12)	S (35)	S (29)	S (29)	S (32)	S (31)	S (33)	S (27)	S (29)	S (35)
DH5α-*bla*_CMY−4_	R (6)	R (6)	S (23)	R (6)	R (6)	R (6)	R (6)	R (10)	S (35)	S (30)	S (35)	S (34)	S (30)	S (33)	S (26)	S (28)	S (35)

*Antibiotic susceptibility tests were performed in duplicate*.

*AMP, Ampicillin; AMC, Amoxicillin; TZP, Piperacillin/Tazobactam; CZ, Cefazolin; CXM, Cefuroxime; CAZ, Ceftazidime; CFM, Cefixime; FEP, Cefepime, IPM, Imipenem; ETP, Ertapenem; MEM, Meropenem; LVX, Levofloxacin; CIP, Ciprofloxacin; GN, Gentamycin; AN, Amikacin; SXT, Cotrimoxazole*.

*S, sensitive; SDD, susceptible-dose dependent; I, intermediate resistance; R, resistant*.

### Mechanisms associated with ertapenem resistance in EC1515

To investigate the mechanisms contributing to ETP resistance in EC1515, the nucleotide sequences and protein levels of OmpA, OmpC, OmpF, and OmpR were analyzed. The western blot results showed that the expression of OmpA, OmpC, and OmpF was similar between EC974 and EC1515 (data not shown), and the results were similar to our previous report (Yan et al., 2010). Sequences of *ompA, ompC, ompF*, and *ompR* were identical between EC974 and EC1515. Moreover, the promoter region and sequence of the chromosome-borne, extended-spectrum AmpC (ESAC) β-lactamase were identical between EC974 and EC1515.

## Discussion

This study characterized genome-wide and phenotypic changes that have occurred in the *E. coli* ST219 clone after nearly 4 months of chronic carriage and antibiotic treatments. Recurrent UTI is one of the most common bacterial infections in older women and children and poses a major clinical challenge worldwide (Guglietta, 2017). Tapiainen et al. showed that recurrent infection isolates were found to form biofilms effectively (Tapiainen et al., 2014). Moreover, the adhesiveness and invasiveness on the cells were significantly higher for *K. pneumoniae* recurrent strains than for initial colonized strains (Lin et al., 2014). Here, we showed that the patient with a recurrent UTI was infected by the same ST219 clone, retrievable as isolates EC974 and EC1515. Moreover, EC974 and EC1515 displayed different colony morphologies, with a rougher colony morphology of EC1515 compared to EC974 (Figure S1). These results indicate evolution of EC974 *in vivo*, with phenotype changes, during adaptation to the host and exposure to antibiotics.

One of the strategies that bacteria adopt to survive stress conditions is a change in morphology. Such changes were observed in bacteria on exposure to toxic organic compounds in *Pseudomonas putida* and *Enterobacter* sp. (Neumann et al., 2005). Temperature was reported to induce morphological changes in *E. coli* (Bennett et al., 1992) and *P. pseudoalcaigenes* (Shi and Xia, 2003). The sequence of antigen 43, an autotransporter in *E. coli* involved in cell aggregation, biofilm, and colony morphology (Kjaergaard et al., 2000a,b), was found to be identical between EC974 and EC1515. In addition, the metabolic pathways for glycolysis and gluconeogenesis determined by KEGG were the same between the two strains (data not shown). Chiang-Ni et al. reported that the hypermucoid variants of *Streptococcus pyogenes* showed a growth-defective phenotype in regular broth culture conditions and are significantly more susceptible to various DNA-damaging treatments when compared with the mucoid variant (Chiang-Ni et al., 2014). Therefore, whether EC1515 displays higher efficacy for cell colonization, biofilm formation, or stress tolerance compare to EC974, thus leading to recurrent UTI, is worth investigating.

Due to the lengthy antibiotic treatment regiments administered to many recurrent UTI patients, this cohort is at an elevated risk of developing multi-drug-resistant infections. Here, we showed that after CRO, CFM, and IMP treatments, EC1515, isolated from the patient with a recurrent UTI infection, was more resistant to TZP, FEP, CPO, and ETP, compared to EC974 (Table 2). Comparative genomic results revealed no new resistance genes acquired by EC1515 during antibiotic treatments (Table 3). We identified *bla*_CMY−111_ in *E. coli*, and demonstrated *bla*_CMY−4_ evolved from *bla*_CMY−111_ by W221R and V238A substitutions within the host. Moreover, *bla*_CMY−4_ showed similar antibiotic resistance patterns (Table 6). Therefore, the acquired resistance genes did not cause resistance to TZP, FEP, CPO, and ETP in EC1515.

We noticed a cluster of small INDELs within the 16-18 kb region of the largest plasmid and the assembly qualities at many of those INDEL loci were low. The low assembly qualities were not the results of low read coverage. On the contrary, the coverage depth of the largest plasmid was about six times greater than the mean depth of the chromosome for both strains. This indicates multiple copies of the largest plasmids in the bacteria, consistent with the results shown in Figure S3. It is possible that there are slightly different versions of that plasmid, resulting in ambiguous assemblies at those small INDEL loci. We also found that the inserted segment on the largest plasmid of strain EC1515 was highly similar to a contig segment of EC974, but that contig was discarded due to its small length (2,061 bp) and low coverage (9.6 X) at the beginning. It is possible that the segment was acquired into the largest plasmid in the strain 1515. The small differences between the two genomes suggest that strain EC1515 really evolved from EC974.

Here, we revealed 12 SNPs and 8 INDELs (quality ≥ 30) in EC1515, compared with EC974, that arose during a 4-month persistent infection within host (Table 5 and Table S1). The mutation rate in EC1515 was higher, when compared with a previous report (1.1 per genome/year) (Reeves et al., 2011). Our results suggest that antibiotic exposure not only imposes a selective challenge to bacterial cells but also accelerates the rate of adaptation by selecting for advantageous mutations, consistent with a previous report (Long et al., 2016). Moreover, our comparative sequence results revealed a SNP of the RecQ protein (repair-related protein) in EC1515 (Table 5). Yang et al. reported that the mutation of RecQ resulted in a mutator phenotype in *E. coli* cells (Yang et al., 2004). However, the mutation rate in EC974 and EC1515 remains to be determined.

ETP has become an important option for the treatment of *E. coli* infections. ETP-resistant *E. coli* strains may appear susceptible to IMP and MEP in routine susceptibility tests (Yan et al., 2010). Combinations of mutations in *envZ, ftsI, mrdA, acrB*, and *acrR* can cause high-level carbapenem resistance, independent of reduced *ompCF* expression in *E. coli* (Adler et al., 2016). However, all known genes involved in ETP resistance in *E. coli* were identical between EC974 and EC1515. We identified SNPs in two regulators (putative transcriptional repressor and BaeR) in EC1515 (Table 5). However, whether the SNPs in EC1515 regulators lead to differential gene expression between EC974 and EC1515 is unclear. The development of next generation sequencing technologies, whole genome sequence and RNA-seq, has provided a novel means to study host-pathogen interaction and bacterial evolution within-host. Therefore, whether the gene expression patterns in EC1515 are different from that in EC974 and thus lead to ETP resistance remains to be clarified by RNA-seq. Moreover, the results raised the possibility that ETP resistance may be caused by a combination of several mutations. In summary, we first identified an IncI1-plasmid carrying *bla*_CMY−111_ in *E. coli* and observed its evolution to *bla*_CMY−4_ *in vivo*.

## Author contributions

C-YK, J-WC, and T-LL designed the study, performed analyses and wrote the manuscript. C-YK and J-WC performed bioinformatics analyses. J-JY and J-JW participated in, coordinated, and supervised the study. All authors approved the final manuscript.

### Conflict of interest statement

The authors declare that the research was conducted in the absence of any commercial or financial relationships that could be construed as a potential conflict of interest.

## References

[B1] Adler M. Anjum M. Andersson D. I. Sandegren L. (2016). Combinations of mutations in *envZ, ftsI*, *mrdA, acrB* and *acrR* can cause high-level carbapenem resistance in *Escherichia coli*. J. Antimicrob. Chemother. 71, 1188–1198. 10.1093/jac/dkv475 26869688

[B2] Al-Agamy M. H. Aljallal A. Radwan H. H. Shibl A. M. (2017). Characterization of carbapenemases, ESBLs, and plasmid-mediated quinolone determinants in carbapenem-insensitive *Escherichia coli* and *Klebsiella pneumoniae* in Riyadh Hospitals. J. Infect. Public Health 11, 64–68. 10.1016/j.jiph.2017.03.010 28462854

[B3] Aziz R. K. Bartels D. Best A. A. DeJongh M. Disz T. Edwards R. A.. (2008). The RAST Server: rapid annotations using subsystems technology. BMC Genomics. 9:75. 10.1186/1471-2164-9-75 18261238 PMC2265698

[B4] Barry A. L. Jones R. N. Thornsberry C. Ayers L. W. Kundargi R. (1985). Imipenem (N-formimidoyl thienamycin): *in vitro* antimicrobial activity and beta-lactamase stability. Diagn. Microbiol. Infect. Dis. 3, 93–104. 10.1016/0732-8893(85)90017-3 3872196

[B5] Bennett A. F. Lenski R. E. Mittler J. E. (1992). Evolutionary adaptation to temperature. I. Fitness responses of *Escherichia coli* to changes in its thermal environment. Evolution 46, 16–30. 10.1111/j.1558-5646.1992.tb01981.x 28564952

[B6] Boyd D. Taylor G. Fuller J. Bryce E. Embree J. Gravel D.. (2015). Complete sequence of four multidrug-resistant MOBQ1 plasmids harboring *bla*GES-5 isolated from *Escherichia coli* and *Serratia marcescens* persisting in a hospital in Canada. Microb. Drug Resist. 21, 253–260. 10.1089/mdr.2014.0205 25545311

[B7] Chiang-Ni C. Zheng P. X. Wang S. Tsai P. J. Kuo C. F. Chuang W. J.. (2014). Invasive hypermucoid variant of group A Streptococcus is defective in growth and susceptible to DNA-damaging treatments. Pathog. Dis. 70, 194–201. 10.1111/2049-632X.12114 24339221

[B8] Clinical and Laboratory Standards Institute (2009). Methods for Dilution Antimicrobial Susceptibility Tests for Bacteria that Grow Aerobically; Approved Standard, 8th Edn. M07-A8. Wayne: Clinical and Laboratory Standards Institute.

[B9] Clinical and Laboratory Standards Institute (2015). Performance Standards for Antimicrobial Susceptibility Testing, Twenty-Third Informational Supplement, M100-S25. Wayne: Clinical and Laboratory Standards Institute.

[B10] Doi Y. Paterson D. L. Adams-Haduch J. M. Sidjabat H. E. O'Keefe A. Endimiani A.. (2009). Reduced susceptibility to cefepime among *Escherichia coli* clinical isolates producing novel variants of CMY-2 beta-lactamase. Antimicrob. Agents Chemother. 53, 3159–3161. 10.1128/AAC.00133-09 19414578 PMC2704647

[B11] Dupont H. Choinier P. Roche D. Adiba S. Sookdeb M. Branger C.. (2017). Structural alteration of OmpR as a source of ertapenem resistance in a CTX-M-15-producing *Escherichia coli* O25b:H4 sequence type 131 clinical isolate. Antimicrob. Agents Chemother. 61:e00014-17. 10.1128/AAC.00014-17 28264855 PMC5404536

[B12] Frost L. S. Leplae R. Summers A. O. Toussaint A. (2005). Mobile genetic elements: the agents of open source evolution. Nat. Rev. Microbiol. 3, 722–732. 10.1038/nrmicro1235 16138100

[B13] Guglietta A. (2017). Recurrent urinary tract infections in women: risk factors, etiology, pathogenesis and prophylaxis. Future Microbiol. 12, 239–246. 10.2217/fmb-2016-0145 28262045

[B14] Ilyas B. Tsai C. N. Coombes B. K. (2017). Evolution of *Salmonella*-host cell interactions through a dynamic bacterial genome. Front. Cell. Infect. Microbiol. 7:428. 10.3389/fcimb.2017.00428 29034217 PMC5626846

[B15] Jacoby G. A. (2009). AmpC beta-lactamases. Clin. Microbiol. Rev. 22, 161–182. 10.1128/CMR.00036-08 19136439 PMC2620637

[B16] Kado C. I. Liu S. T. (1981). Rapid procedure for detection and isolation of large and small plasmids. *J*. Bacteriol. 145, 1365–1373. 10.1128/jb.145.3.1365-1373.1981 PMC217141 7009583

[B17] Kao C. Y. Udval U. Huang Y. T. Wu H. M. Huang A. H. Bolormaa E.. (2016). Molecular characterization of extended-spectrum beta-lactamase-producing *Escherichia coli* and *Klebsiella* spp. isolates in Mongolia. J. Microbiol. Immunol. Infect. 49, 692–700. 10.1016/j.jmii.2015.05.009 26194952

[B18] Kao C. Y. Yan J. J. Lin Y. C. Zheng P. X. Wu J. J. (2017). Complete genome sequence of carbapenem-resistant *Klebsiella pneumoniae* strain 1756, isolated from a pus specimen. Genome Announc. 5:e00066-17. 10.1128/genomeA.00066-17 28360152 PMC5374226

[B19] Kent W. J. (2002). BLAT–the BLAST-like alignment tool. Genome Res. 12, 656–664. 10.1101/gr.229202 11932250 PMC187518

[B20] Kjaergaard K. Schembri M. A. Hasman H. Klemm P. (2000a). Antigen 43 from *Escherichia coli* induces inter- and intraspecies cell aggregation and changes in colony morphology of *Pseudomonas fluorescens. J*. Bacteriol. 182, 4789–4796. 10.1128/JB.182.17.4789-4796.2000 PMC111355 10940019

[B21] Kjaergaard K. Schembri M. A. Ramos C. Molin S. Klemm P. (2000b). Antigen 43 facilitates formation of multispecies biofilms. *Environ*. Microbiol. 2, 695–702. 10.1046/j.1462-2920.2000.00152.x 11214802

[B22] Larsen M. V. Cosentino S. Rasmussen S. Friis C. Hasman H. Marvig R. L.. (2012). Multilocus sequence typing of total-genome-sequenced bacteria. J. Clin. Microbiol. 50, 1355–1361. 10.1128/JCM.06094-11 22238442 PMC3318499

[B23] Lin W. H. Kao C. Y. Yang D. C. Tseng C. C. Wu A. B. Teng C. H.. (2014). Clinical and microbiological characteristics of *Klebsiella pneumoniae* from community-acquired recurrent urinary tract infections. Eur. J. Clin. Microbiol. Infect. Dis. 33, 1533–1539. 10.1007/s10096-014-2100-4 24756209

[B24] Lin Y. T. Huang Y. W. Huang H. H. Yang T. C. Wang F. D. Fung C. P. (2016). *In vivo* evolution of tigecycline-non-susceptible *Klebsiella pneumoniae* strains in patients: relationship between virulence and resistance. Int. J. Antimicrob. Agents 48, 485–491. 10.1016/j.ijantimicag.2016.07.008 27575728

[B25] Long H. Miller S. F. Strauss C. Zhao C. Cheng L. Ye Z.. (2016). Antibiotic treatment enhances the genome-wide mutation rate of target cells. Proc. Natl. Acad. Sci. U.S.A. 113, E2498–E2505. 10.1073/pnas.1601208113 27091991 PMC4983809

[B26] Ma L. Siu L. K. Lin J. C. Wu T. L. Fung C. P. Wang J. T.. (2013). Updated molecular epidemiology of carbapenem-non-susceptible *Escherichia coli* in Taiwan: first identification of KPC-2 or NDM-1-producing *E. coli in Taiwan*. BMC Infect. Dis. 13:599. 10.1186/1471-2334-13-599 24354657 PMC3878139

[B27] Neumann G. Veeranagouda Y. Karegoudar T. B. Sahin O. Mausezahl I. Kabelitz N.. (2005). Cells of *Pseudomonas putida* and *Enterobacter* sp. adapt to toxic organic compounds by increasing their size. Extremophiles 9, 163–168. 10.1007/s00792-005-0431-x 15765202

[B28] Nordmann P. Mammeri H. (2007). Extended-spectrum cephalosporinases: structure, detection and epidemiology. Future Microbiol. 2, 297–307. 10.2217/17460913.2.3.297 17661704

[B29] Nordmann P. Naas T. Poirel L. (2011). Global spread of Carbapenemase-producing *Enterobacteriaceae*. Emerging Infect. Dis. 17, 1791–1798. 10.3201/eid1710.110655 22000347 PMC3310682

[B30] Pacheco A. B. Soares K. C. de Almeida D. F. Viboud G. I. Binsztein N. Ferreira L. C. (1998). Clonal nature of enterotoxigenic *Escherichia coli* serotype O6:H16 revealed by randomly amplified polymorphic DNA analysis. *J. Clin*. Microbiol. 36, 2099–2102. 10.1128/jcm.36.7.2099-2102.1998 PMC104989 9650973

[B31] Pires J. Taracila M. Bethel C. R. Doi Y. Kasraian S. Tinguely R.. (2015). *In vivo* evolution of CMY-2 to CMY-33 beta-lactamase in *Escherichia coli* sequence type 131: characterization of an acquired extended-spectrum AmpC conferring resistance to cefepime. Antimicrob. Agents Chemother. 59, 7483–7488. 10.1128/AAC.01804-15 26392491 PMC4649241

[B32] Pop M. Phillippy A. Delcher A. L. Salzberg S. L. (2004). Comparative genome assembly. Brief. Bioinform. 5, 237–248. 10.1093/bib/5.3.237 15383210

[B33] Reeves P. R. Liu B. Zhou Z. Li D. Guo D. Ren Y.. (2011). Rates of mutation and host transmission for an *Escherichia coli* clone over 3 years. PLoS ONE. 6:e26907. 10.1371/journal.pone.0026907 22046404 PMC3203180

[B34] Seiffert S. N. Hilty M. Kronenberg A. Droz S. Perreten V. Endimiani A. (2013). Extended-spectrum cephalosporin-resistant *Escherichia coli* in community, specialized outpatient clinic and hospital settings in Switzerland. J. Antimicrob. Chemother. 68, 2249–2254. 10.1093/jac/dkt208 23759671

[B35] Shi B. Xia X. (2003). Morphological changes of *Pseudomonas pseudoalcaligenes* in response to temperature selection. Curr. Microbiol. 46, 120–123. 10.1007/s00284-002-3824-4 12520367

[B36] Tapiainen T. Hanni A. M. Salo J. Ikäheimo I. Uhari M. (2014). *Escherichia coli* biofilm formation and recurrences of urinary tract infections in children. Eur. J. Clin. Microbiol. Infect. Dis. 33, 111–115. 10.1007/s10096-013-1935-4 23996047

[B37] Temkin E. Adler A. Lerner A. Carmeli Y. (2014). Carbapenem-resistant *Enterobacteriaceae*: biology, epidemiology, and management. Ann. N. Y. Acad. Sci. 1323, 22–42. 10.1111/nyas.12537 25195939

[B38] Viberg L. T. Sarovich D. S. Kidd T. J. Geake J. B. Bell S. C. Currie B. J.. (2017). Within-host evolution of *Burkholderia pseudomallei* during chronic infection of seven Australasian cystic fibrosis patients. MBio 8:e00356–17. 10.1128/mBio.00356-17 28400528 PMC5388805

[B39] Yan J. J. Wu J. J. Lee C. C. Ko W. C. Yang F. C. (2010). Prevalence and characteristics of ertapenem-nonsusceptible *Escherichia coli* in a Taiwanese University Hospital, 1999 to 2007. Eur. J. Clin. Microbiol. Infect. Dis. 29, 1417–1425. 10.1007/s10096-010-1020-1 20700614

[B40] Yang H. Wolff E. Kim M. Diep A. Miller J. H. (2004). Identification of mutator genes and mutational pathways in *Escherichia coli* using a multicopy cloning approach. Mol. Microbiol. 53, 283–295. 10.1111/j.1365-2958.2004.04125.x 15225322

[B41] Zankari E. Hasman H. Cosentino S. Vestergaard M. Rasmussen S. Lund O.. (2012). Identification of acquired antimicrobial resistance genes. J. Antimicrob. Chemother. 67, 2640–2644. 10.1093/jac/dks261 22782487 PMC3468078

