# Supplementary material for: Comparative Genomics of Escherichia coli Sequence Type 219 Clones From the Same Patient: Evolution of the IncI1 blaCMY-Carrying Plasmid in Vivo
Source: Front Microbiol. 2018 Jul 9;9:1518. doi: 10.3389/fmicb.2018.01518 (PMC6046403; doi:10.3389/fmicb.2018.01518)
Supplement: Supplementary file 1 [file Data_Sheet_1.doc]

**Supplementary materials**

**DNA techniques**

Mini Qiagen columns and a QiaAmp DNA extraction kit (Qiagen, Valencia, CA, USA) were used for chromosomal DNA extraction. The primer used for RAPD-PCR analysis was primer 1254 (5’-CCGCAGCCAA-3’) (Akopyanz et al., 1992; Pacheco et al., 1998). RAPD-PCR was carried out according to the manufacturer's instructions using Taq polymerase (Promega, Madison, WI, USA). The RAPD-PCR program consisted of 4 cycles (5 min at 94°C, 5 min at 36°C, and 5 min at 72°C), followed by 30 cycles (1 min at 94°C, 1 min at 36°C, and 2 min at 72°C). A final 10-min extension was performed at 72°C.

**Plasmid profile analysis**

Plasmid numbers and sizes present in parental isolates and transconjugants were verified by Kado and Liu's methods (Kado & Liu, 1981).

**References**

1. Akopyanz, N., Bukanov, N.O., Westblom, T.U., Kresovich, S., and Berg, D.E. (1992). DNA diversity among clinical isolates of *Helicobacter pylori* detected by PCR-based RAPD fingerprinting. *Nucleic Acids Res*. 20, 5137–5142.
2. Kado, C.I., and Liu, S.T. (1981). Rapid procedure for detection and isolation of large and small plasmids. *J*. *Bacteriol*. 145, 1365–1373.
3. Pacheco, A.B., Soares, K.C., de Almeida, D.F., Viboud, G.I., Binsztein, N., and Ferreira, L.C. (1998). Clonal nature of enterotoxigenic *Escherichia coli* serotype O6:H16 revealed by randomly amplified polymorphic DNA analysis. *J*. *Clin*. *Microbiol*. 36, 2099–2102.

**Fig. S1**

**
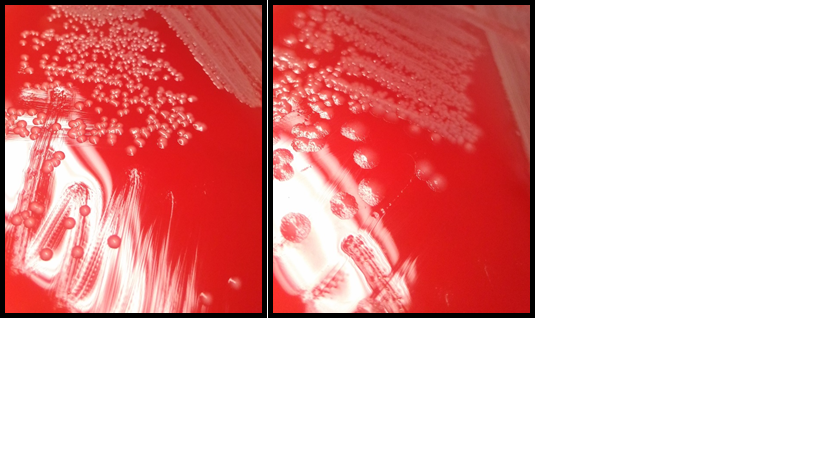
**

**Fig. S1. Colony morphologies on sheep blood agar. EC974 (left) has a smooth colony morphology, while EC1515 (right) has a rougher colony morphology.**

**Fig S2.**

**
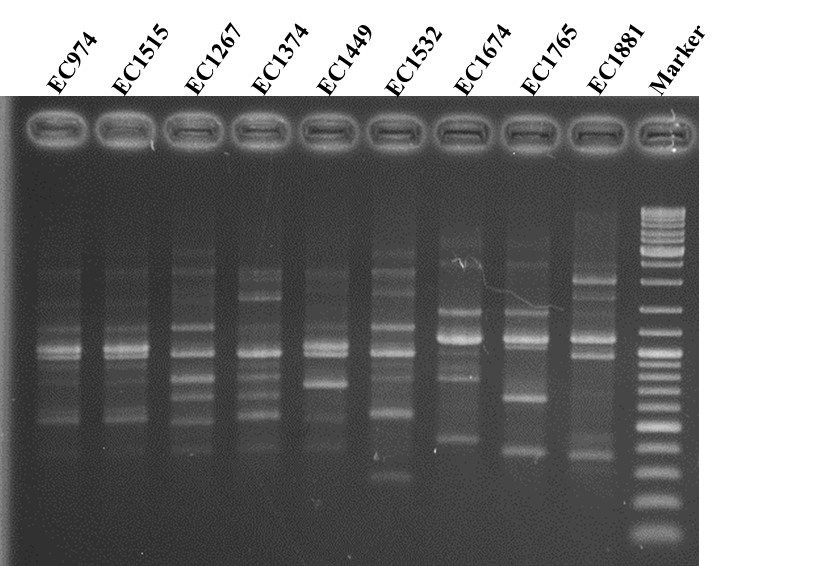
**

**Fig. S2. Agarose gel of RAPD-PCR profiles generated with primer 1254.** Strains EC1267, EC1374, EC1449, EC1532, EC1674, EC1765, and EC1881, collected from patients with urinary tract infections in the same hospital, were randomly selected and used as internal controls to show the discriminatory ability of primer 1254. Marker: GeneRuler^TM^ DNA Ladder Mix (Fermentas, Thermo Fisher Scientific). The experiment was performed in duplicate.

**Fig. S3**

**
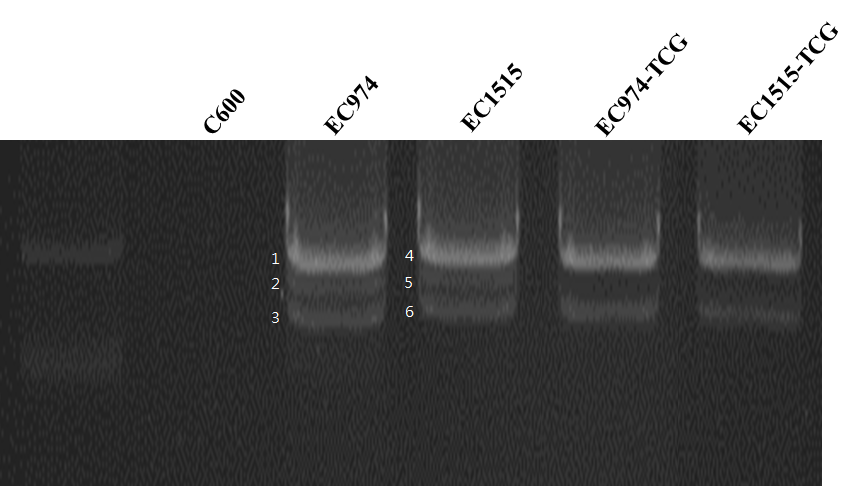
**

**Fig. S3. Profiles of plasmids of the five *E. coli* strains.** *E. coli* C600 was used as negative control. 1, pEC974-1; 2, pEC974-2; 3, pEC974-3; 4, pEC1515-1; 5, pEC1515-2; 6, pEC1515-3. The experiment was conducted in duplicate.

**Table S1. SNPs and INDELs within the uniquely aligned segments of the two assemblies**

| Contig | EC974 | | EC1515 | |
| --- | --- | --- | --- | --- |
|  | Locus | Base | Locus | Base |
| Chr. | 1378463 | C | 1377490 | G |
| Chr. | 1524239 | T | 1523265 | - |
| Chr. | 1588773 | A | 1587799 | G |
| Chr. | 1857685 | C | 1856711 | T |
| Chr. | 2230098 | T | 2229124 | G |
| Chr. | 2264204 | C | 2263229 | - |
| Chr. | 2309203 | - | 2308229 | C |
| Chr. | 2352437 | T | 2351462 | - |
| Chr. | 2414261 | A | 2413286 | T |
| Chr. | 2514423 | G | 2513448 | C |
| Chr. | 3073707 | A | 3072732 | T |
| Chr. | 3260965 | G | 3259990 | A |
| Chr. | 3432619 | - | 3431645 | C |
| Chr. | 4466967 | G | 4465993 | T |
| Chr. | 4566979 | T | 4566004 | - |
| P1 | 1392 | T | 1392 | A |
| P1 | 16651 | T | 16650 | - |
| P1 | 16720 | C | 16718 | - |
| P1 | 16729 | C | 16726 | - |
| P1 | 16744 | C | 16740 | - |
| P1 | 16847 | C | 16842 | - |
| P1 | 16907 | G | 16901 | - |
| P1 | 16947 | G | 16940 | - |
| P1 | 16992 | C | 16984 | - |
| P1 | 17001 | G | 16992 | - |
| P1 | 17075 | C | 17065 | - |
| P1 | 17171 | G | 17160 | - |
| P1 | 17173 | A | 17161 | - |
| P1 | 17177 | - | 17166 | T |
| P1 | 17178 | - | 17168 | C |
| P1 | 17225 | A | 17739 | - |
| P1 | 17228-17229 | CC | 17741 | - |
| P1 | 17235 | C | 17746 | - |
| P1 | 17259-17261 | AAG | 17769 | - |
| P1 | 17264 | T | 17771 | - |
| P1 | 17266-17268 | GGG | 17772 | - |
| P1 | 17271 | C | 17774 | - |
| P1 | 17302 | G | 1780 | - |
| P1 | 17308 | C | 17809 | - |
| P1 | 17396 | G | 17896 | - |
| P1 | 17459 | G | 17958 | - |
| P1 | 17496 | C | 17994 | - |
| P1 | 17544 | G | 18041 | - |
| P1 | 17636 | - | 18134 | T |
| P1 | 49297 | T | 49468 | A |
| P1 | 49349 | T | 49520 | C |
| P2 | 36578-36629 | CAGAAATGCTCCACGTACTCGGCCCCTTCCGGCGTGAAGGCGGCATCGAGCG | 36577 | - |
|  | | | | |
